# Spatial extrapolation of cadmium concentration in terrestrial mosses using multiple linear regression model predictions across French biogeographical regions

**DOI:** 10.1007/s11356-025-35985-5

**Published:** 2025-02-06

**Authors:** Jérémy Lamouroux, Caroline Meyer, Sébastien Leblond, Isabelle Albert

**Affiliations:** 1 MIA-Paris-Mathématiques Et Informatique Appliquées, INRAE-AgroParisTech-Université Paris-Saclay, 22 Place de L’Agronomie, 91477 Palaiseau, France; 2 PatriNat (OFB-MNHN), 12 Rue Buffon, 75005 Paris, France

**Keywords:** Forest mosses, Heavy metal, Linear model, Spatial extrapolation, Prediction map, Biomonitoring

## Abstract

The French Moss Survey employs forest mosses as indicators to monitor the deposition of atmospheric substances, notably focusing on cadmium (Cd), a known carcinogenic and contributor to respiratory illnesses. This comprehensive study encompasses 55 variables to understand Cd accumulation in terrestrial mosses in France. These variables include moss species, tree cover, biogeographical markers, land use area, proximity to road and rail networks, soil concentration of Cd and atmospheric concentration and deposition of Cd using a physical model. The response variable undergoes a complementary log–log transformation to constrain prediction values within the maximum Cd content in mosses. We have built a regression model to improve predictions, considering the impacts of covariates in France. This model retains biogeographical effects, leading to data segmentation into four distinct biogeographical zones: Atlantic, Continental, Mediterranean and Alpine. Subsequently, zone-specific regression models are explored to refine predictions and consider the impacts of covariates specific to each region, such as those related to railways and roads of the Mediterranean zone. Our biogeographical models effectively mitigate spatial correlation issues and yield accurate predictions, as evidenced by the leave-one-out cross-validation assessment. Compared to ordinary kriging map, the regression prediction maps highlight the contributions of certain covariates, such as the EMEP atmospheric transport model, to areas with high Cd concentrations. Furthermore, these maps exhibit new areas with high (resp. low) Cd concentrations due to high (resp. low) values of the covariates.

**Supplementary Information:**

The online version contains supplementary material available at 10.1007/s11356-025-35985-5.

## Introduction

Many metallic elements in ionic or particulate form are present in the atmosphere. These elements become toxic above a certain threshold (Nordberg et al. 2007) and cause major health problems for ecosystems and humans (Gurjar et al. 2010). Among these elements, cadmium (Cd), which is carcinogenic and triggers respiratory disease (Andujar et al. 2010), accumulates in organisms (Zhang and Reynolds 2019). Moreover, Cd is biomagnified in food webs (Butt et al. 2018) and leads to a loss of biodiversity (Zwart et al. 2010). Most Cd impact studies focus on high concentrations. However, chronic exposure to low concentrations can also increase the risk of cancer (Lequy et al. 2023) and death from heart and respiratory diseases (Lequy et al. 2019). Therefore, assessing the spatial distribution of Cd levels is necessary, especially in rural areas where contamination is considered low compared to urban areas (Vieille et al. 2021).

Few monitoring stations in France measure atmospheric and deposition levels of cadmium (Cd), and those that do are primarily located in urban or peri-urban areas. These measurement sites remain limited due to technical challenges and operating costs. Although these stations meet regulatory requirements for monitoring atmospheric metals, more is needed to provide comprehensive predictions, particularly at a national level where monitoring is sparse. Modelling plays a crucial role in compensating for this lack of coverage. In Europe, for example, cadmium (Cd) deposition and atmospheric concentrations are modelled by the European Monitoring and Evaluation Programme (EMEP) using an atmospheric transport model (Ilyin et al. 2013; Travnikov and Ilyin 2005), which relies on data about emissions and meteorological conditions from the concerned countries.

Since the 1960s, terrestrial mosses have been used to monitor atmospheric metals, including Cd, at fine scales. This allows for higher sampling densities than conventional deposition measurements (Gusev et al. 2009). Terrestrial mosses morphologies, the absence of roots and a developed vascular system make them excellent sensors of atmospheric contaminants as they depend on atmospheric deposition (Bates 1992). The concentrations of atmospheric Cd are commonly considered proxies for atmospheric deposition. However, it is essential to understand the sources and environmental factors modulating Cd accumulation by mosses (Lequy et al. 2023).

Several studies have demonstrated that Cd concentrations in mosses are influenced by various factors, including Cd deposition modelled by EMEP (Schröder et al. 2019), the percentage of land use determined by the CORINE Land Cover (CLC) (Schröder et al. 2014) and the presence of forests or urban areas (Lequy et al. 2022) or the Cd concentration of the soil on which the mosses grow (Taeprayoon et al. 2023). Moss species and forest cover also play a role in determining metal concentrations (Holy et al. 2009). More importantly, these relationships are contingent. There are three principal approaches to addressing this issue. The first relies on explicative models identifying the most significant covariates explaining metal concentrations in mosses (Lequy et al. 2022). The second approach is based on analyses to understand the links between each component using Spearman correlations, Bartlett’s tests and Dunn’s tests (Harmens et al. 2012; Boquete et al. 2020). The last approach uses mapping based on regression models or decision trees (Harmens et al. 2011; Schröder et al. 2017). The importance of certain variables becomes apparent in some research works (Lequy et al. 2022), which focused on urban land in France, identifying forest and urban land use as significant variables. Another study on forestry land in France highlighted moss species, atmospheric concentration and deposition as significant variables (Lequy et al. 2017a, b). Interestingly, studies in Germany (Schröder et al. 2019) revealed different significant variables including population density and distance to federal highways. The concept of regionalisation (Vieille et al. 2021) was demonstrated by the varying effects of variables when comparing moss sampling in urban land in two French cities: Paris and Lyon. This regionalisation phenomenon underscored the link between the study area and the relevant covariates. Overall, these assessments contribute to our understanding of the environmental impact of moss accumulation and the distribution of metal concentrations within a region.

This study aims to understand and establish the spatial representation of Cd background levels across mainland France. Cd levels in mosses are influenced by atmospheric deposition, land use and environmental factors such as forest cover, population density and proximity to urban areas or highways. These factors likely vary regionally, reflecting a phenomenon of regionalisation, where different environmental and anthropogenic covariates are significant in distinct areas. Using multiple regression models, we can identify these significant covariates and predict the spatial distribution of Cd in mosses with more precision than traditional methods like ordinary kriging. We first seek to analyse and compare multiple regression models to identify the most relevant environmental and anthropogenic variables affecting Cd concentrations in mosses across France and its biogeographical zones. We then aim to predict the spatial distribution of Cd from these models. To do so, data collected from 445 sites of the BRAMM (Biosurveillance des Retombées Atmosphériques Métalliques par les Mousses) network were analysed.

## Materials and methods

### Measurements Cd in forest mosses across France

#### Study sites and moss sampling

**Fig. 1:**
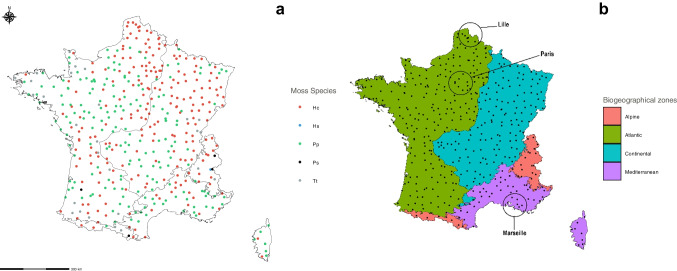
**a** The 445 observed forest sites (BRAMM sites), depending on the moss species. **b** The four biogeographical zones, defined by the official boundaries established in the REGBIOFR layer (Roekaerts 2012). Hc, *Hypnum cupressiforme* Hedw.; Pp, *Pseudoscleropodium purum* (Hedw.) M.Fleisch.; Ps, *Pleurozium schreberi* (Willd. ex Brid.) Mitt.; Tt, *Thuidium tamariscinum* (Hedw.) Schimp.; Hs, *Hylocomium splendens* (Hedw.) Schimp

During the BRAMM 2016 campaign, moss samples were collected from 445 forested sites evenly spread across France. These sites represent background pollution levels. Moss samples were collected following the standardised protocol of ICP Vegetation (Monitoring ICP Manual, 2020). In brief, a single moss species was collected from areas distant from any known local pollution sources. Each site covered an area of 2500 m^2^ and was located below tree canopies. One of five different moss species was collected for each site to obtain samples from 445 sites: *Hypnum cupressiforme* Hedw., 1801 (Hc, 227 samples), *Pseudoscleropodium purum* (Hedw.) M.Fleisch., 1923 (Pp, 186 samples), *Thuidium tamariscinum* (Hedw.) Schimp., 1852 (Tt, 27 samples), *Pleurozium schreberi* (Willd. ex Brid.) Mitt., 1869 (Ps, 4 samples), and *Hylocomium splendens* (Hedw.) Schimp., 1852 (Hs, 1 sample) (Fig. 1a), respectively named Hc, Pp, Tt, Ps and Hs hereafter. The five species could be distinguished in the field using morphological criteria that could be observed with a magnifying glass (magnification × 10; lens diameter 21 mm). For each sample, 10 fragments of the same moss species carpets were collected. *Hypnum cupressiforme* was always collected on dead wood, while other species were collected on humus (composed solely of decomposing organic matter) or soil (composed of a mixture of inorganic and organic matter). Each sample was stored in plastic and brought to the laboratory within the same week. At the laboratory, samples were carefully cleaned from all dead material and attached litter. The whole moss shoot was kept. Samples were dried at room temperature before grinding with an automatic non-polluting titanium grinder (Pulverisette®14, Fritsch, Germany). All necessary precautions were taken to avoid contamination while working with trace levels, including using powder-free gloves, plastic and ceramic pincers and scissors.

#### Analysis of the collected moss samples and quality assurance

A total of 250 mg of homogeneous powder was dried for 48 h at 24 °C and sent to USRAVE (INRAE-Centre de Bordeaux) for analysis. The powder was mineralised with a mixture of HF/HNO_3_/H_2_O_2_ (concentrations expressed at 103 °C after accounting for water loss on a subsample). Cadmium in mosses was analysed by inductively coupled plasma mass spectrometry (ICP-MS) by the USRAVE (INRAE-Centre de Bordeaux) (limit of quantification was 0.02 µg/g; analytical uncertainty is 20%). The device used by the USRAVE was an Agilent 7700 × spectrometer.

The ICP-MS was calibrated by external calibration, using multi-element solutions produced every 6 months. The internal calibration procedure involved the addition of the standard. USRAVE uses the NIST 1547 (peach leaves) to validate their results as an internal Certified Reference Material for trace elements (CRM). We had 3 other CRMs analysed namely, NIST 1573a (tomato leaves), as well as European moss survey programme reference materials—Moss M2 and Moss M3—were processed with each batch of samples. The Moss M2 and Moss M3 standards were powders of *Pleurozium schreberi* from two Finnish sites (Berg et al. 1997).

### Variables of interest

Table 1 presents the 55 constructed variables from information collected at each site, which could explain the variability of Cd concentration in mosses. In this study, the substrate from which the moss was taken was not retained as a variable due to a bias in the protocol as the sampling method created a strong link between substrate and moss species. These 55 variables correspond to 65 covariates, which can be categorised as follows:

i). Categorical covariates encompass 3 variables which are the following: the moss species collected corresponding to 5 covariates (Hc, Hs, Pp, Ps and Tt), the biogeographical zones corresponding to 4 covariates (Atlantic, continental, Mediterranean and Alpine), and the different tree canopies on the sampling site corresponding to 4 covariates (deciduous, coniferous, mixed deciduous tree and mixed coniferous tree).
ii). A quantitative covariate encompasses 1 variable which corresponds to 1 covariate, the altitude of the sampling location (in metres).
iii). Quantitative covariates involve 36 variables, i.e. 36 covariates, land use percentage covariates, expressed as the percentage of land use area within buffer zones of 1 km, 5 km, 10 km and 15 km from the sampling sites, calculated using the 2018 CLC database (with a resolution of 100 × 100 m).
iv). Quantitative covariates also involve 8 variables, i.e. 8 covariates, distance-related covariates, which measure the distances in metres to roads and railways within the same buffer zones (1 km, 5 km, 10 km and 15 km).
v). Two quantitative covariates derived from the EMEP model include two separate measures represented by 6 variables. Firstly, the atmospheric deposition of Cd in (g/km^2^/year) was calculated by summing deposits over the 1, 3, 6, 9 or 12 months preceding the collection period, moving backwards to derive 5 variables, i.e., 5 covariates (EMEP_dep1, EMEP_dep3, EMEP_dep6, EMEP_dep9, EMEP_dep12). Secondly, the atmospheric concentration of Cd in (µg/m^3^/year), modelled in 2016 with a resolution of 0.1° × 0.1°, corresponds to 1 variable, i.e., 1 covariate.
vi). A quantitative covariate, represented by 1 variable, i.e. 1 covariate, of Cd soil concentration value (mg/kg^2^) modelled in 2018 for the period 2000–2010 by the RMQS network (Gis Sol) in a grid cell of 2 × 2 km.

**Table 1:** List of 55 constructed variables and their description as potential predictor of Cd accumulation in mosses in 2016

Variable		Code	Source	Calculating variables with CLC codes	Number and name of covariates^¤^
Moss species		**Moss**	**BRAMM**^**a**^		**5: Hc, Pp, Ps, Tt, Hs**
Tree cover		**Tree**	**BRAMM**^**a**^		**4: deciduous, coniferous, mixed deciduous tree, mixed coniferous tree**
Biogeographical zone		**Biogeo**	**INPN**^**b**^		**4: Continental, Atlantic, Mediterranean, Alpine**
Altitude		**Altitude**	**Copernicus**^**c**^		**1**
Forested land uses (1, 5, 10, and 15 km radius)		Forest_*	**CLC**^**d**^	**244 + 311 + 312 + 313 + 323 + 324**	**4**
Urban land uses (1, 5, 10, and 15 km radius)		Urban_*	**CLC**^**d**^	**111 + 112 + 122 + 141 + 142**	**4**
Industrial land uses (1, 5, 10, and 15 km radius)		Industrial_*	**CLC**^**d**^	**121 + 123 + 124 + 131 + 132 + 133**	**4**
Pasture land uses (1, 5, 10, and 15 km radius)		Pasture_*	**CLC**^**d**^	**231**	**4**
Agricultural land uses (1, 5, 10, and 15 km radius)		Agricultural_*	**CLC**^**d**^	**211 + 212 + 213 + 241 + 242 + 243**	**4**
Fruit land uses (1, 5, 10, and 15 km radius)		Fruits_*	**CLC**^**d**^	**221 + 222 + 223**	**4**
Vegetation land uses (1, 5, 10, and 15 km radius)		Vegetation_*	**CLC**^**d**^	**321 + 322 + 333**	**4**
Water land uses (1, 5, 10, and 15 km radius)		Water_*	**CLC**^**d**^	**335 + 411 + 412 + 511 + 512**	**4**
Sea land uses (1, 5, 10, and 15 km radius)		Sea_*	**CLC**^**d**^	**331 + 421 + 422 + 423 + 521 + 522 + 523**	**4**
Railway (1, 5, 10, and 15 km radius)		Railway_*	**CLC**^**d**^		**4**
Road (1, 5, 10, and 15 km radius)		Road_*	**CLC**^**d**^		**4**
Cd air deposition (1, 3, 6, 9, and 12 month)		**EMEP_dep**^+^	**EMEP**^**e**^		**5**
Cd air concentration		**EMEP_air**	**EMEP**^**e**^		**1**
Cd soil total		**RMQS_tot**	**RMQS**^**f**^		**1**

^**a**^Biosurveillance des Retombées Atmosphériques Métalliques par les Mousses, https://bramm.mnhn.fr

^b^L'Inventaire National du Patrimoine Naturel, https://inpn.mnhn.fr

^c^Copernicus, https://www.copernicus.eu/fr

^**d**^Corine Land Cover, https://land.copernicus.eu/pan-european/corine-land-cover

^e^European Monitoring and Evaluation Programme, https://www.emep.int/

^f^Réseau de Mesures de la Qualité des Sols, https://www.gissol.fr/le-gis/programmes/rmqs-34

^*^The length of radius in km

^**+**^The number of months summed

^¤^The total of covariates 65

### Exploration of biogeographical zones

France can be delineated into various zones based on regional, departmental, city or economic criteria. The primary consideration in such divisions is often the geographical extent. In our study, we adopted the division of Metropolitan France into four biogeographical zones, as defined in the Habitats Directive (92/43/EEC). We used the REGBIOFR layer which defined the official boundaries of biogeographical regions (Fig. 1b). These divisions, endorsed by the Habitats Committee in 2012 (Roekaerts 2012), provided a systematic framework for understanding and managing biodiversity within each region.

The division of France into biogeographical zones was a crucial aspect of our study methodology. This regionalized approach enhanced our ability to capture nuanced environmental factors and their influence on metal accumulation within mosses across Metropolitan France.

### Multiple regression model

#### Complementary log–log link

Multiple regression models are widely utilised in data analysis, assuming a linear relationship between response variables, such as Cd concentration in mosses, and explanatory covariates, as described in the “Variables of interest” section. These models offer interpretability through coefficients, indicating the strength and direction of relationships. Linear models also help identify outliers and influential observations. To achieve a more normal distribution of the response variable and align predictions with realistic thresholds, we explored transforming the response variable.

Our method used a complementary log–log (CLL) linear model; the inverse of the Gumbel cumulative distribution function yields the CLL link function (Shim et al. 2023). The model is the following:

$$ln(ln\left(\frac{C{d}_{max}}{{Y}_{i}}\right))=\alpha +\sum\nolimits_{j=1}^{N}{\beta }_{j}{X}_{i}^{j}+{\epsilon }_{i},$$

where $ln$(.) is the natural logarithm function, ${Y}_{i}$ is the Cd concentration in mosses *i* in (*µg/g*), ${Cd}_{\text{max}}$ fixed to 1.5 µg*/g* according to the maximal expected value of Cd in mosses, $\alpha$ and ${\beta }_{j}$ are the intercept and slope regression coefficients, respectively, $\sum_{j=1}^{N}{X}_{i}^{j}$ are the sum of *N* independent covariates ${X}^{j}$ of interest and ${\epsilon }_{i}\sim N\left(0,{\sigma }^{2}\right)$, where *N*(0, ${\sigma }^{2}$) is a normal distribution with a mean of 0 and a variability of ${\sigma }^{2}$.

#### Covariate selection

In the initial step, we filtered out 55 variables (excluding the 3 qualitative variables) with more than 95% of zero values within each biogeographical zone. For example, 95% of the 445 observations had a 0 value for the covariate Marin in the Continental zone. This ensured that the indication provided by the remaining covariates was rare and pertinent, as an overwhelming presence of zeros might render the information irrelevant. Consequently, the number of covariates decreased for each zone. For instance, in the case of the regression model encompassing France, the covariate ‘Fruits’ with a buffer of a 1-km radius was removed due to this criterion.

Following this initial step, we employed the Spearman correlation to investigate collinearity problems between covariates. This non-parametric measure assesses the strength and direction of the relationship between two ranked variables. Spearman correlation values range from − 1 to + 1, with + 1 indicating a perfect positive correlation (Schober et al. 2018). If the Spearman correlation between multiple variables exceeded 0.75, we addressed this by considering two options. Firstly, if the same covariates with different buffers exhibit high correlation (e.g. ‘Forest_1’, ‘Forest_5’, ‘Forest_10’ and ‘Forest_15’), we retained the one with the smallest buffer size (in this case, ‘Forest_1’). Secondly, if there was a correlation between two distinct covariates, such as ‘Forest_1’ and ‘Culture_1’, and the correlation value exceeded 0.75, we retained the covariate with a lower correlation and a separate buffer, here ‘Forest_1’.

To produce the actual selection of variables, i.e. to retain in the model only those variables that influence the response, the Cd concentration in the mosses, we employed a backward-forward selection approach using the Akaike information criterion (AIC), balancing goodness of fit and model complexity. Minimising the AIC value ensured the selection of the most appropriate regression model, effectively reducing the dimensionality while retaining the most influential explicative covariates for our response variable.

#### Residual spatial correlation

After selecting the best model, we aimed at investigating whether the spatial correlation of observations remained when covariates are not considered. Unaccounted environmental factors may have contributed to similarities between observations. Thus, determining whether this spatial correlation persisted after introducing selected covariates was essential. The Spatial Correlation Moran Index (Banerjee et al. 2003) is a metric designed to assess whether similar values tend to cluster or disperse across a geographic area and can be used for this purpose. Positive values of the Moran Index, calculated on the response or residuals of the multiple regression, indicate clustering. This suggests that similar values are grouped together. In contrast, negative values imply dispersion, indicating that similar values are spread apart. A value of 0 suggests the absence of a spatial pattern.

### Prediction maps

#### Drawing up concentration maps

It is important to extrapolate regression model predictions to comprehensively assess continuous and fine-tuned territorial variations in Cd concentrations across France. We established a spatial grid covering France with a resolution of 2 × 2 km to match the size of the moss sampling area, which was a circular area of 2500 m^2^. Predictors were calculated by inverting the multiple regression CLL linear models, providing predictions $\widehat{y}(s)$ (expressed as Hc mosses under deciduous cover tree) at each point (s) of the spatial grid, totalling 139,673 points across France. This calculation required obtaining the values of each covariate retained in the models, sourced from relevant databases covering the territory.

We also produced a map of the standard deviations of each prediction (obtained using the predict function from terra R package, which approximates this variance using the delta method). This was useful for visualising the uncertainty associated with the calculated prediction.

Also, to assess the contribution of our covariates to our predictions, we compared our prediction maps (point prediction and standard deviation) with maps obtained using the classic ordinary kriging (OK) method, which relied solely on observations (response) and their spatial covariance to obtain predictions (Cressie 1990). The observations underwent a logarithmic transformation to improve normality.

#### Investigation of the predictive performance

The precision of the extrapolation methods was assessed using the leave-one-out cross-validation (LOOCV) technique (Wilcox et al. 2012). LOOCV is a widely used approach for comparing the accuracy of extrapolation methods. It involves systematically excluding each sampling site. Subsequently, the value at the excluded site was estimated based on the prediction at the site calculated with the model excluding the site. This iterative process was repeated until all sampling sites have prediction values, resembling an extreme case of k-fold cross-validation, where *k* equals the total data points in the dataset. Test points were used to calculate the root mean squared error (RMSE), which quantified the average difference between the predictions of the dataset and the observed values. This measurement helped assess extrapolation performance (Stone 1974; Tyagi et al. 2022):

$$\text{LOOCV }RMSE=\sqrt{\sum\nolimits_{i=1}^{M}\frac{{(y}_{i}-{\widehat{y}}_{i}{)}^{2}}{M}},$$

where at site *i,* ${y}_{i}$ is the Cd concentration observed, ${\widehat{y}}_{i}$ is the concentration prediction fitted without ${y}_{i}$ and *M* is the total number of observations in the model. The lower the LOOCV value, the better the model predictions match the observed data.

All analyses were conducted using R version 4.2.2. Spatial data manipulation and analysis were performed using the ‘terra’ package (version 1.7–39). Linear regression models were fitted using the ‘lm’ function, and model selection was based on the Akaike information criterion (AIC), calculated using the ‘AIC’ function. Predictions were generated using the ‘predict’ function, and spatial visualisations were created using the ‘ggplot’ function.

## Results

### Cadmium concentration in CRMs and BRAMM mosses

The assessment of our 3 CRMs (Table 2) from the 15 series of analyses carried out during the 2016 campaign validated the accuracy of our results. There was no difference between the mean Cd concentration, including its standard deviation, associated with the reference values and those obtained from our analyses.

**Table 2:** Elementary statistics of Cd concentrations analysed in reference materials (SRM 1573a, Moss M2, and Moss M3, *n* = 15) and in 445 moss samples taken during the 2016 BRAMM campaign

	References materials **			Cd (BRAMM 2016)
	Moss M2	Moss M3	NIST 1573a	
Number of values	15	15	15	445
Minimum	0.433	0.0931	1.350	0.029
1st quartile	0.453	0.107	1.440	0.099
Median	0.460	0.111	1.470	0.135
3rd quartile	0.477	0.113	1.505	0.199
Maximum	0.558	0.127	1.640	1.160
Mean	0.468	0.111	1.473	0.174
Standard deviation	0.028	0.009	0.079	0.138
Coefficient of variation (%)	6	8	5	79
Ratio max/min	1	1	1	40
Atypical high threshold*	0.513	0.123	1.602	0.349
Number of typical threshold values	1	2	1	28

^*^Atypical high threshold = 3rd quart. + 1,5 * (3rd quart. − 1st quart.)

^**^Values of reference materials: Moss M2 = 0.454 ± 0.019 µg/g; Moss M3 = 0.106 ± 0.005 µg/g; NIST 1573a = 1.52 ± 0.04 µg/g

### Model and map encompassing France

#### Model selection

We started by analysing a model encompassing France, utilising a dataset of 445 observations and 55 variables corresponding to 65 covariates. We removed variables with excessive zeros and high collinearity, as detailed in the “Materials and methods” section. Lastly, we retained 27 variables (Table 3) corresponding to 37 covariates.

**Table 3:** List of variables before and after the backwards-forwards Akaike index criterion (AIC) selection on the complementary log–log linear model in France and each biogeographical zone. Full model = variables selected after removing those for which more than 95% of the values were equal to 0, then after selection by Spearman correlation; relevant = variables selected after backward-forward AIC selection; total of variables = total number of variables selected with the number of significant variables in brackets (*p* < 0.05)

Variable	Code	Selection									
		France (*n* = 445)		Atlantic zone (*n* = 220)		Continental zone (*n* = 147)		Mediterranean zone (*n* = 49)		Alpine zone (*n* = 29)	
		Full model	Relevant	Full model	Relevant	Full model	Relevant	Full model	Relevant	Full model	Relevant
Moss species	**Moss**^**a**^	**✓**	**✓**	**✓**	**✓**	**✓**	**✓**	**✓**		**✓**	**✓**
Tree cover	**Tree**^**a**^	**✓**	**✓**	**✓**	**✓**	**✓**	**✓**	**✓**	**✓**	**✓**	**✓**
Biogeographical zone	**Biogeo**^**b**^	**✓**	**✓**								
Altitude	**Altitude**^**c**^	**✓**		**✓**		**✓**	**✓**	**✓**	**✓**	**✓**	**✓**
Forested land uses (1, 5, 10, and 15 km radius)	**Forest_**^**¤d**^	**1, 5**		**1, 5**	**1**	**1, 5**	**1, 5**	**1, 5**	**1, 5**	**1, 5**	**1, 5**
Urban land uses (1, 5, 10, and 15 km radius)	**Urban_**^**¤d**^	**1, 5**	**5**	**1, 5**	**5**	**1, 5**	**5**	**1, 5**		**1, 5**	**1, 5**
Industrial land uses (1, 5, 10, and 15 km radius)	**Industrial_**^**¤d**^	**5, 10**	**5**	**5, 10**		**5**		**5**		**5, 10**	**5,10**
Pasture land uses (1, 5, 10, and 15 km radius)	**Pasture_**^**¤d**^	**1, 5**		**1, 5**	**1, 5**	**1, 5**	**5**	**1, 5**	**1**	**1, 5**	**1, 5**
Agricultural land uses (1, 5, 10, and 15 km radius)	**Agricultural_**^**¤d**^	**1, 5**	**1**	**5**		**1, 5**	**5**	**1, 5**	**1, 5**	**1, 5**	**1, 5**
Fruits land uses (1, 5, 10, and 15 km radius)	**Fruits_**^**¤d**^	**5, 10**		**5, 10**	**5, 10**	**1, 5**	**5**	**1, 5**	**5**	**10**	**10**
Vegetation land uses (1, 5, 10, and 15 km radius)	**Vegetation_**^**¤d**^	**1, 5**		**5, 10**		**1, 5**	**5**	**1, 5**	**1, 5**		
Water land uses (1, 5, 10, and 15 km radius)	**Water_**^**¤d**^	**5, 10**		**5, 10**		**5, 10**		**5, 10, 15**	**5, 10, 15**	**5, 10**	**5, 10**
Sea land uses (1, 5, 10, and 15 km radius)	**Sea_**^**¤d**^	**10**	**10**	**5, 10**	**10**			**5**	**5**		
Railway (1, 5, 10, and 15 km radius)	**Railway_**^**¤d**^	**5**		**5, 10**		**5**	**5**	**5, 10**	**5, 10**	**5**	**5**
Road (1, 5, 10, and 15 km radius)	**Road_**^**¤d**^	**5**		**5**		**5**		**5, 10, 15**	**5, 10**	**5, 10**	**5, 10**
Cd air deposition (1, 3, 6, 9, and 12 month)	**EMEP_dep**^**+e**^	**1, 3**		**1, 3**		**1**	**1**	**1, 3**		**1**	**1**
Cd air concentration	**EMEP_air**^**e**^	**✓**	**✓**	**✓**	**✓**	**✓**		**✓**	**✓**	**✓**	**✓**
Cd soil total	**RMQS_tot**^**f**^	**✓**	**✓**	**✓**		**✓**		**✓**	**✓**	**✓**	**✓**
Total of variables		**27**	9 (7*)	**27**	10 (8*)	**23**	12 (5*)	**29**	20 (11*)	**22**	22 (3*)
AIC		**167.81**		**12.79**		**87.90**		**−9.47**		**181.27**	

^a^Biosurveillance des Retombées Atmosphériques Métalliques par les Mousses, https://bramm.mnhn.fr/

^b^L'Inventaire National du Patrimoine Naturel, https://inpn.mnhn.fr

^c^Copernicus, https://www.copernicus.eu/fr

^d^Corine Land Cover, https://land.copernicus.eu/pan-european/corine-land-cover

^e^European Monitoring and Evaluation Programme, https://www.emep.int/

^f^Réseau de Mesures de la Qualité des Sols, https://www.gissol.fr/le-gis/programmes/rmqs-34

^¤^The length of radius in km

^+^The number of months summed

^*^Covariates with significant *p*-value < 0.05

The AIC backward-forward selection retains 9 relevant variables for modelling France (Table 3): moss species, tree cover, biogeographical zone, urban land uses with a buffer of 5 km, industrial land uses with a buffer of 5 km, agricultural land uses with a buffer of 1 km, sea land uses with a buffer of 10 km, Cd soil total RMQS and Cd air concentration EMEP. The selected model reaches an AIC value of 167.81. Only 7 of these covariates demonstrate a significant *p*-value < 0.05, as shown in Table 4.

**Table 4:** Significant covariates (*p* < 0.05) and their effect (negative or positive) on the model in France and each biogeographical zone. Moss Hs, Moss Pp, Moss Ps, and Moss Tt = covariates associated with the variable moss species and expressed relatively to the Hc moss species (Hs, *Hylocomium splendens*; Pp, *Pseudoscleropodium purum*; Ps, *Pleurozium schreberi*; Tt, *Thuidium tamariscinum*; Hc, *Hypnum cupressiforme*)*.* Biogeo ATL, Biogeo CON, Biogeo MED = covariates associated with the variable Biogeographical zone and expressed relatively to the Alpine zone (ATL, Atlantic zone; CON, Continental zone; MED, Mediterranean zone). Coniferous trees, mixed coniferous tree, mixed deciduous tree = covariates associated with the variable tree cover and expressed relatively to the deciduous trees

Code	France		Atlantic zone		Continental zone		Mediterranean zone		Alpine zone	
	Effect	*p*_value	Effect	*p*_value	Effect	*p*_value	Effect	*p*_value	Effect	*p*_value
Intercept	** − **	** < 0.001**	** − **	** < 0.001**	** + **	** < 0.05**				
Moss Hs										
Moss Pp	** − **	** < 0.001**	** − **	** < 0.001**	** + **	** < 0.001**				
Moss Ps	** − **	** < 0.05**							** − **	** < 0.05**
Moss Tt	** − **	** < 0.001**	** − **	** < 0.01**						
Biogeo ATL	** + **	** < 0.05**								
Biogeo CON										
Biogeo MED										
Coniferous trees	** + **	** < 0.001**	** + **	** < 0.05**	** + **	** < 0.001**				
Mixed coniferous tree	** + **	** < 0.001**	** + **	** < 0.001**	** + **	** < 0.05**	** − **	** < 0.05**		
Mixed deciduous tree										
Altitude					** − **	** < 0.05**				
Forest_1			** + **	** < 0.05**						
Forest_5							** − **	** < 0.05**		
Urban_1										
Urban_5										
Industrial_5										
Industrial_10										
Pasture_1							** + **	** < 0.01**		
Pasture_5			** − **	** < 0.05**	** + **	**0.05**				
Agricultural_1	** − **	**0.01**								
Agricultural_5							** − **	**0.05**		
Fruits_5										
Fruits_10			** + **	** < 0.05**						
Vegetation_1							** + **	** < 0.01**		
Vegetation_5					** + **	** < 0.05**	** − **	** < 0.01**		
Water_10										
Water_15										
Sea_5							** − **	** < 0.05**		
Sea_10	** − **	** < 0.001**	** − **	** < 0.05**						
Railway_5							** + **	** < 0.01**		
Railway_10							** − **	** < 0.05**		
Road_5							** − **	** < 0.01**		
Road_10										
EMEP_dep1									** − **	**0.05**
EMEP_air	** + **	** < 0.001**	** + **	** < 0.001**			** + **	** < 0.05**	** + **	**0.05**
RMQS_tot	** − **	** < 0.05**								

Three significant qualitative covariates influence the concentration of Cd in mosses: moss species, tree cover and biogeographical zone. These covariates highlight the importance of moss species, the specific forest cover where the mosses grow and the biogeographical zone in which they are situated. Two quantitative covariates exhibit high significance levels (*p* < 0.001): Sea_10, with a negative effect, and EMEP_air, with a positive effect. Conversely, EMEP_air indicates that as Cd air concentration increases, Cd concentrations in mosses also rise. On the contrary, the percentage of sea land uses around the moss collection area are associated with lower Cd concentrations in mosses. Another quantitative covariate, Agricultural_1, shows statistical significance at *p* < 0.01, with a negative effect. Higher percentages of agricultural land uses around the moss collection area are associated with lower Cd concentrations in mosses. Finally, the last significant covariate, RMQS_tot_1, reaches statistical significance at *p* < 0.05, and its influence is positively oriented (Table 4). When the concentration of Cd in the soil is high at the location where the moss has been collected, the concentration of Cd in the moss is higher. Urban_5 and Industrial_5 are non-significant but remain in the model because they reduce the AIC and improve the model’s goodness of fit compared to a model without them.

The application of Moran’s index, as described in the “Material and methods” section, assesses whether the model with 9 variables fully accounts for spatial effects or if residual spatial autocorrelation remains after accounting for these covariates. Table 5 indicates that residual spatial autocorrelation persists, suggesting that the covariates do not fully explain the spatial patterns observed. Note that Moran’s index value is divided by 2 in our analysis.

**Table 5:** Moran’s index (*I*-statistics) before and after considering covariates in the models. Atlantic, Continental, Mediterranean, Alpine = biogeographical zone. NS is not significant (*p* > 0.05)

	Without considering covariates		Considering covariates	
	*I*-statistics	*p*-value	*I*-statistics	*p*-value
France	0.307	*p* < 0.001	0.154	*p* < 0.05
Atlantic zone	0.424	*p* < 0.001	0.011	*p* < 0.05
Continental zone	0.091	*p* < 0.001	− 0.010	NS
Mediterranean zone	0.064	*p* < 0.001	0.201	NS
Alpine zone	0.476	*p* < 0.001	− 0.181	NS

#### Prediction maps

**Fig. 2:**
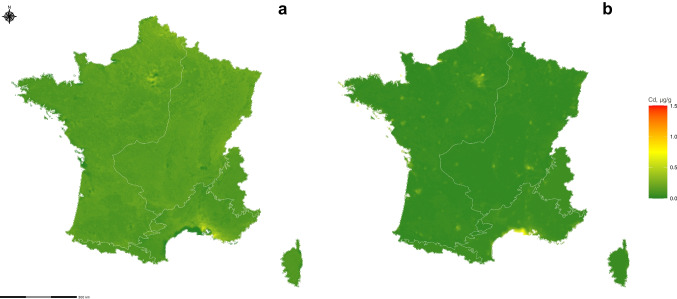
**a** Predictions of Cd concentration accumulated by *Hypnum cupressiforme* Hedw. under deciduous cover tree (µg/g) from the selected 9 covariate model. White lines, border of the biogeographical zones. **b** Standard deviation of the prediction from the selected 9 covariate model

**Fig. 3:**
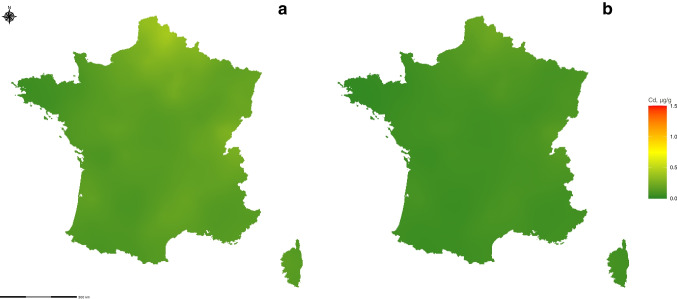
**a** Ordinary kriging of Cd concentration in mosses (µg/g). **b** Standard deviation of ordinary kriging

We constructed a prediction map of Cd concentration throughout France using the 9 retained variables. Figure 2a and b depict the extrapolation of Cd concentration with moss Hc under deciduous forest cover, along with standard deviation maps. The white borders delineating biogeographical zones indicate the relevance of the variable to the model. The standard deviation maps illustrate prediction accuracy, with higher or lower predictions corresponding to higher standard deviations. Supplementary data provide additional maps for other moss species and forest cover types. Figure 3a displays the ordinary kriging of Cd concentration in mosses, while Fig. 3b represents the standard deviation of ordinary kriging values.

Our spatial analysis reveals elevated predictions of Cd concentrations along the border with Belgium and in northern France, particularly in proximity to Paris. Another zone with higher Cd concentration predictions has also been identified near Marseille in southern France. Conversely, within the Mediterranean zone, a distinct low prediction of Cd concentrations is observed. The standard deviation map highlights significant variability, with pronounced high- and low-concentration prediction fluctuations. Notably, the southern region of France exhibits substantial variability, corresponding to areas of elevated predictions. A similar pattern is observed near Paris.

It is interesting to compare the previous map of the model’s prediction across France with the ordinary kriging of Cd concentration in mosses (Fig. 3a). Both indicate higher concentrations in mosses near Lille and Paris, but an important difference is observed near Marseille. The regression models’ predictions are notably higher near Marseille and are related to the highest values modelled by EMEP. The regression model’s prediction map also reveals a lower coastline contamination level attributed to the sea covariate within a 10-km buffer. The maps covering France (Fig. 2a and Fig. 3a) highlight the possibility of high Cd concentrations in mosses near the Belgian border and the city of Lille. However, the standard deviation of these predictions is high (Fig. 2b and Fig. 3b). The standard deviation map shows greater variability where the high and low concentration predictions lie.

Table 6 gives the LOOCV of the 9-variable model over France (0.296) calculated zone by zone. Additionally, the LOOCV for ordinary kriging is reported as 0.491 over France. Comparing the two methods regarding the LOOCV criterion (Table 6), our maps demonstrate predictions closer to the observations, instilling confidence in the other’s predictions in areas lacking observation data for comparison.

**Table 6:** Leave-one-out cross-validation (LOOCV) with root-mean-square deviation (RMSE) to compare the accuracy of models in France and each biogeographical zone. Atlantic, Continental, Mediterranean, and Alpine

	LOOCV RMSE	LOOCV RMSE
	Encompassing France	Biogeographical zone
France	**0.2961672**	**0.3620656**
Atlantic zone	**0.2542687**	**0.2467906**
Continental zone	**0.3290859**	**0.3239615**
Mediterranean zone	**0.3766574**	**0.2872906**
Alpine zone	**0.3250785**	**0.9370611**

### Models and maps by biogeographical zone

Given the statistical significance of the biogeographical zone covariate and the persistence of spatial residuals, we explore more refined models within each biogeographical zone.

#### Model selection

Table 3 presents the full model for each biogeographical zone, including the number of observations and covariates in the model after removing variables with too many zeros and those that are too collinear. Additionally, Table 3 displays the relevant model selected based on the smallest AIC criterion and the significant covariates in these models. Table 4 provides the significance levels of the covariates.

In the Atlantic zone, a model built with 220 observations using backward-forward AIC selection retains 10 relevant variables, including 8 with significant associations with the response variable. Two qualitative variables, moss species and tree cover, are retained due to significant differences from the intercept (Hc mosses and deciduous trees). Pp mosses (*p* < 0.001) and Tt mosses (*p* < 0.01) exhibit significant differences from Hc mosses under deciduous trees. The mixed coniferous tree (*p* < 0.001) and coniferous tree (*p* < 0.05) demonstrate distinctions from Hc mosses in the same context. These findings emphasise the influence of moss species and forest cover on moss collection in the region. Quantitative covariates, EMEP_air, show high statistical significance (*p* < 0.001) with a positive effect, consistent with the broader model encompassing France. Additionally, covariates Forest_1 and Fruits_10 positively influence the outcome (*p* < 0.05), while Pasture_5 and Sea_10 exhibit negative effects at the same significance level. Including covariates such as Fruits_10, which are absent in the French model, provides additional insights into the dynamics of the Atlantic zone.

In the Continental zone, based on 147 observations, the backward-forward AIC selection retains 12 variables in the model. Only 5 have a significant effect on the Cd concentration. Significant qualitative covariates include moss species and tree cover, which are retained in the model. Quantitative covariates such as Vegetation_5, Pasture_5 and Altitude are significant (*p* < 0.05). Two novel covariates, previously unselected by models encompassing France and the Atlantic region, emerge as notable contributors: Vegetation_5, which exhibits a positive effect and altitude, which manifests a negative impact. Geographical considerations can elucidate the inclusion of these covariates. Areas of higher altitude, such as the Jura and Massif Central, substantiate the introduction of the altitude covariate. The higher we are, the less Cd there is. Conversely, Vegetation_5 is linked to the prevalence of natural pasturelands, heaths and shrublands within the continental zone. Additionally, extensive agricultural landscapes in this region are associated with Pasture_5. These two covariates increase the concentration of Cd. The selection of these covariates in the Continental zone reflects a logical and context-specific rationale, aligning with this territory’s distinctive environmental characteristics and land use patterns. Consequently, the absence of specific covariates, like Sea_10, is expected, as they are unrelated to the specific attributes of this region.

In the Mediterranean zone, based on 49 observations, we applied the backward-forward AIC selection, which retains 20 variables. 11 of them have a significant effect on the Cd concentration. The qualitative covariates corresponding to the tree cover are significant, while moss species do not show significance in this zone. New quantitative covariates are selected and significant in this model: Pasture_1, Vegetation_1, Water_15, Sea_5 and Road_5. These covariates, previously unselected in models across France, the Atlantic and the Continental zones, contribute new information specific to the Mediterranean zone. EMEP_air, Railway_5, Vegetation_5, agricultutral_5 and Forest_5 are the same as the previous region and France (Table 4). The positive effect of Railway_5, contrasting with its effect in the Continental zone, is potentially linked to factors such as Mediterranean forest fires (Hubert et al 2002). Moreover, Railway_10 and Road_5, two road-related covariates, demonstrate statistically significant coefficients with a negative impact on Cd concentration, likely due to the extensive railway and road networks in the Mediterranean zone. Several other covariates, including Vegetation_1, Water_15, Sea_5, Vegetation_5, Agricultural_5 and Forest_5, are consistent with the environmental characteristics unique to the Mediterranean zone. The covariate associated with atmospheric conditions, EMEP_air, consistently exhibits a positive effect in this region, as observed in other zones where it is included. Among the highly significant covariates (*p* < 0.01), Paturage_1, Vegetation_1, Ferre_5 and Routier_5 have positive coefficients, while Vegetation_5 has a negative coefficient.

In the Alpine zone, our observations are constrained by a limited sample size of 29 against the number of variables (22 variables retained by the AIC selection). However, only three covariates are found to affect Cd concentration significantly. The qualitative covariate of moss species is notably significant. In quantitative covariates, only two show statistical significance: EMEP_dep1 and EMEP_air. EMEP_dep1 has a negative effect, while EMEP_air has a positive effect across most regions in France, except for the Continental zone, where it has no significant impact. This suggests that EMEP_air contributes to increased Cd concentration across the various regions.

As for encompassing France, we examine the correlation between observed Cd concentrations before and after accounting for covariates in biogeographical zones to assess their impact on spatial correlation in Cd mosses concentrations (response variable). Table 5 provides a summary of these findings.

We are pleased to see that covariates effectively address spatial correlation in Cd concentrations in mosses across three biogeographical zones (Continental, Mediterranean, Alpine).

On the other hand, in the Atlantic zone, the regression model without covariates yields a highly significant Moran *I* Index (*p* < 0.05) and an *I* statistic of 0.424. Conversely, the model incorporating 10 variables leads to a less significant *p*-value and an *I* statistic of 0.011. Notably, including covariates in this zone substantially account for the spatial correlation of Cd concentrations observed in mosses. This improvement surpasses that observed for the encompassing French model, for which the spatial correlation is only halved. The predictions of our maps will likely be completely independent.

#### Prediction map

**Fig. 4:**
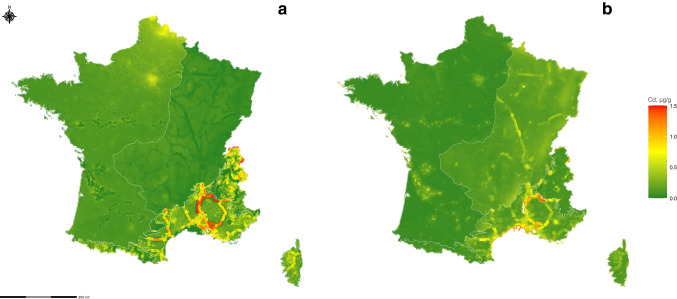
**a** Predictions of Cd concentration accumulated by *Hypnum cupressiforme* Hedw. under deciduous cover tree (µg/g) from each model of biogeographical zones. White lines, border of the biogeographical zones. **b** Standard deviation of the prediction from each model of the biogeographical zone

From each final model selected in each zone, we construct a Cd prediction map for the biogeographical zone (and a map of the standard deviations of the predictions). Figure 4a depicts the composite map of these regional predictions, culminating in a comprehensive depiction of France based on distinct biogeographical models. This is a much more nuanced map of France in each biogeographical zone than the map encompassing France (Fig. 2a) and the kriging (Fig. 3a).

The Cd prediction in the Atlantic zone shows higher Cd concentrations near Lille (1 µg/g Cd) and Paris (0.65 µg/g Cd) regions in northern France. A similar pattern emerges when we compare these results with the model encompassing France (0.65 µg/g) near Lille and Paris. However, the model covering the Atlantic zone offers a more nuanced perspective, incorporating new covariates that enhance the depth of information and our understanding of the observed patterns. By comparing our results visually with the kriging map, we note that areas near Lille and Paris are also present. Our model does indeed find this spatial similarity. The Atlantic zone shows the highest standard deviation near Paris, corresponding to higher Cd prediction values in mosses. This phenomenon is not found near Lille along the Belgian border, which has more observations (Fig. 1a). The standard deviation map shows greater variability where predictions of high and low concentrations are found.

The Continental zone shows slightly lower Cd concentrations along railway lines, as the covariate is present in the model but not significant (the standard deviation of these predictions is high in these areas, as evidenced in Fig. 4b). We can see from Fig. 3a that the railway zones are absent if we visually compare our results for this zone with the kriging map. However, the covariate coefficient is not significant in the model (Table 3).

In the Mediterranean zone, very high Cd concentrations are predicted near railways within a 5-km buffer due to the significant positive effect of this covariate in the model and the important presence of railways in the region (many predictions in the 2 $\times$ 2-km grid have a very high railways covariate value). In addition, we can observe the influence of the negative effect of the Sea_5 covariates along the west coast near the city of Marseille with lower Cd concentration predictions. The highest values modelled by EMEP_air are also represented near Marseille. EMEP_air is significant in the model, explaining the high values found in this area. Thus, the Mediterranean zone map offers a new perspective compared with the other maps (Fig. 2a and Fig. 3a), highlighting the possibility of high Cd concentrations in mosses along railway lines. However, the standard deviation of these predictions is high.

The Alpine zone highlights a significant impact of EMEP_air, which is present in the regression model with a positive effect. Let us compare this result visually with the map of encompassing France. We note that the impact of the EMEP_air covariate is absent from the predictions constructed on the encompassing model for France in this zone, whereas the kriging map reveals some of this effect.

Table 6 presents the LOOCV scores of the biogeographical model calculated on a zone-by-zone basis and France using the different models. Employing separate models for each zone for the Atlantic, Continental and Mediterranean zones yields superior LOOCV scores compared to using a single model for all of France. However, in the Alpine zone, the LOOCV score for the model encompassing all of France (0.328) is lower than the zone-specific LOOCV score (0.937). This discrepancy is observed since the model has nearly as many covariates as observations, and removing an observation to calculate an LOOCV criterion probably makes it unreliable. Consequently, the overall LOOCV score for France, calculated using different models for each zone (0.362), surpasses that obtained with a single model for the entire country (0.2961). Nevertheless, the LOOCV score for France using the zone-by-zone approach remains lower than that obtained through the kriging method (0.491).

## Discussion

### Covariates selected and comparison with literature

Quantitative covariates such as urban, agricultural, sea or forest land use were categorised from the CLC datasets (Nickel et al. 2017). We used numerous transformations based on the CLC (Table 1) to leverage the most relevant covariates strengthening their link to Cd concentrations in mosses. For instance, while the CLC subclass ‘grove’ was denoted by the 222 and 223 codes, we introduced another category labelled ‘fruits’ including these two codes as well as the 221 code which corresponded to the ‘wine-growing’ label.

The present study sought to analyse relevant covariates such as moss species, urban land uses, altitude and Cd air concentration EMEP modelled data, which were assessed in prior research by Schröder et al. (2010), Nickel et al. (2017), Harmens et al. (2014) and Holy et al. (2009). Notably, although EMEP encompasses Cd deposition and air concentrations, previous studies predominantly focused on EMEP Cd deposition. In this analysis, we included deposition and air concentration metrics, revealing that air concentration emerged as the preferred covariate based on its significant *p*-values, except for the Continental zone. A study considering all of Europe (Holy et al. 2009) has modelled these two EMEP covariates. In this study, the covariate selection retained the EMEP deposition covariate as significant. Another relevant study focusing solely on Germany (Schröder et al. 2019) also identified EMEP deposition as a significant covariate. This result could be due to multiple geographical and biological differences in the predefined territories of Europe, Germany and France. Our study globally examined a wider range of variables (22 in the Alpine zone to 29 in the Atlantic zone) compared to previous studies by Schröder et al. (2010) and Nickel et al. (2017), investigating 11 and 9 variables, respectively. In line with prior research, the EMEP coefficient emerged as a significant predictor in our analysis. However, we noted spatial dependence in the EMEP coefficient, exhibiting varying values across different regions of France. The observed spatial variability underscores the importance of conducting further investigations into the complex relationships between EMEP and Cd concentrations in moss. Moreover, Cd air concentrations were distributed across three French biogeographical zones, while Cd deposition was evidenced only in the Alpine and Continental zones.

Between 2010 and 2021, major sources of Cd were identified as originating from industries, transportation, human activities, agricultural practices and waste, as reported in Citepa (2023). Our model established a link between Cd concentrations in moss in France and the following selected covariates: urban, industrial and agricultural land uses. Parallel studies conducted by Lequy et al. (2022) and Schröder et al. (2017), Schröder et al. (2010)) also highlighted the relevance of these covariates, particularly the urban and agricultural influences. Furthermore, the divergence from the biogeographical model was apparent in our study across distinct Atlantic, Continental, Mediterranean and Alpine zones. Consequently, specific patterns emerged when examining the biogeographical zones. The Atlantic zone showed associations with relevant factors such as fruit (relevant to wine-growing), which had a positive effect, and pasturelands, which has a negative effect. The Mediterranean zone exhibited stronger correlations with transportation networks (railway and road infrastructure). Moreover, qualitative covariates such as moss species, tree cover and biogeographical zone consistently influence the French model. These outcomes resonate with a prior study (Lequy et al. 2017a, b), highlighting the consistent selection of similar moss species and quantitative covariates like Cd air concentration, industrial land uses and urban land uses.

### Selection criteria

Diverse modelling approaches, such as linear regression, linear mixed models and decision trees, were employed to assess these covariates. Several alternative methodologies were considered, such as classification and regression trees (CART) (Holy et al. 2009) and linear regression with *R*^2^ selection (Harmens et al. 2011). The difference lies in the selection constraints. For instance, while *R*^2^ selection significantly improved model goodness of fit, it did not penalise the model size (i.e. more covariates led to better goodness of fit). Our selection process followed a three-step methodology: starting with Spearman correlation (Schröder et al. 2019), followed by expert input to carefully limit the number of variables while retaining relevant information for our model. Subsequently, we employed the AIC (Lequy et al. 2017a, b) using a backward-forward selection technique, ensuring an unbiased model selection process. We reduced dimensionality and minimised the AIC while retaining non-significant covariates to enhance model goodness of fit. Given the large number of covariates at the start of the AIC selection (around thirty), an alternative might have been to leverage a ridge or lasso method. The ridge or lasso method is used in the context of a high number of variables relative to the size of the number of observations (Tibshirani 1996). We therefore applied these methods in our case study. However, these approaches were either too sparse (e.g. lasso did not retain any covariates in the biogeographical zone) or too inclusive (e.g. ridge does not eliminate any covariates in the biogeographical zone). Thus, we found the AIC to be a suitable compromise between model parsimony and goodness of fit, consistent with existing literature.

Some studies on mosses, such as those conducted by Holy et al. (2009) and Schröder et al. (2019), often overlooked the spatial dimension in their models when selecting and applying covariates. Our investigation identified spatial correlation in Cd concentrations across France, a factor discarded during covariate selection in previous studies. To address this issue, we initially partitioned France into biogeographical zones, which mitigated the problem. As a result, residual correlation in 3 out of 4 models was negligible after considering the covariates. A spatial statistical model could be applied to fully assess the spatial pattern of the data (Dormann et al. 2007).

### Relevance of biogeographical zone division

As mentioned previously, the partition of France in the biogeographical zone has already demonstrated the benefits of introducing relevant covariates in each zone to elucidate the spatial correlation over France. Another advantage was shown when evaluating the leave-one-out cross-validation (LOOCV) criterion to assess the predictive performance of each zone. In three out of four zones, these criteria demonstrated improved performance compared to the analysis encompassing the entirety of France. Although this zoning strategy enhanced model performance, its reliance on a limited number of observations raised concerns regarding the reliability of measures such as LOOCV. This limitation was particularly evidenced in the Alpine zone, which comprised 29 observations and 22 variables selected via AIC backward-forward selection. An alternative partitioning of West Europe, known as the Ecological Land Classification of Europe (ELCE) (Hornsmann et al. 2008), provided a division into 40 distinct regions, which gave us a finer breakdown of France (more than 4 zones). Such a delimitation may prove to be beneficial in scenarios where a larger volume of observations is available for analysis.

### Benefits and drawbacks of multiple linear regression models to make prediction maps

Applying linear models for predicting Cd concentration in mosses produced cartographic representations with enhanced contrast and more comprehensive information content than kriging maps. Each significant coefficient in our model played a crucial role in shaping the structure of the resulting map. Interpretation of these maps was facilitated by examining the estimated coefficients associated with the relevant covariates (Schröder et al. 2019). For instance, the Mediterranean zone became discernible in our model which may not be the case in kriging maps. This is due to the inclusion of significant covariates such as EMEP_air or Sea_5 in our model. The presence of specific covariates, such as Sea_5 reduced Cd concentration in the Mediterranean zone. This phenomenon can be attributed to the sea salt effects (Renaudin et al. 2018) but also to the direction of the prevailing winds in this region. In the French metropolis, the winds come mainly from the land, even though the sites are close to the sea, while in Corsica, they are arriving from the sea. Furthermore, establishing a connection between the modelisation of atmospheric Cd concentration (EMEP_air) and the concentrations observed in mosses added valuable insight. It has to be noted that the validity of these predictive maps relied on the validation of moss measurements within these prediction zones. However, regression models exhibited limitations (Zuur et al. 2009), especially when dealing with a limited number of observations, which can decrease their reliability. These models may face challenges in accommodating outliers, leading to the generation of extreme values. Additionally, predicting a zone posed specific challenges due to variations in environmental conditions. The transition from collecting mosses in areas under tree cover, away from pollution sources, to predicting in urban zones introduces a stark environmental contrast. This disparity can challenge achieving coherent predictions, potentially leading to extreme values. To address this issue, we considered transforming the response variable, imposing constraints that aligned with the characteristics of the collected mosses. This transformation helped to ensure that the predictions remained within a meaningful and realistic range of values, accounting for the distinct conditions encountered in different zones. Employing a complementary log–log transformation was a viable strategy to circumvent these issues. Despite confining the predictive values within the Cd concentration range, this transformation limited our ability to derive mean-based maps directly. As a result, we generated maps based on medians. To assess the variability of our predictive models, we also examined the standard deviation derived from the model predictions. Analysing the standard deviation offered valuable insights to quantify the model’s ability to capture and explain the observed data.

Furthermore, we generated a spatial grid with a 2 × 2-km resolution to calculate our predictions based on a multiple regression complementary log–log model. The model was constructed using concentrations collected from mosses situated under forest cover, away from local sources of pollution across France. Our predictions spanned 139,673 pixels on the grid throughout the country, with a varying number of pixels in different biogeographical zones. Notably, this grid enabled predictions in urban zones close to pollution sources. The choice of this fine-scale grid allowed us to produce detailed maps that convey richer information due to the inclusion of covariates, as opposed to traditional kriging methods.

The LOOCV criterion was a valuable tool for looking at the prediction performance. It involved systematically removing one observation from the model and then predicting it using the remaining data. This process assessed the ability of a model to predict new data points. However, it did not necessarily reflect its performance within the observed data space. Moreover, caution is needed when extrapolating beyond the observed data, especially in cases where the prediction grid includes geographic areas significantly different from the observed data, such as urban regions. Further measurements within the territory can provide full validation of our predictions.

## Conclusion

Using 445 sites in a multiple linear regression model to predict and visualise Cd background levels in mainland France provided valuable insights into the factors influencing Cd concentrations across the French territory. The resulting models also allowed for the creation of informative prediction maps on a fine scale of the territory. It highlighted the relationships between Cd concentrations in mosses and various anthropogenic environmental factors, such as railroads, and the concentrations of airborne Cd accumulated by mosses. Our method produced more detailed maps compared to those generated by ordinary kriging. Adding a spatial effect could increase its accuracy in order to improve this prediction and mapping model.

## Supplementary Information

Below is the link to the electronic supplementary material.

Supplementary file1 (PDF 111 KB)

Supplementary file2 (PDF 197 KB)

## Data Availability

Data on Cd concentrations in mosses are publicly available on request from Sébastien Leblond (sebastien.leblond@mnhn.fr) or Caroline Meyer (caroline.meyer@mnhn.fr). All models used for this work are publicly available. The localisation of the data can be accessed via the following dataset on the Global Biodiversity Information Facility (GBIF): https://www.gbif.org/dataset/166cbacd-528a-4a84-b1db-0177b50ac946. Information regarding the Cd measurements is available at: https://bramm.mnhn.fr/cadmium-cd/.
