# Supplementary material for: Spatial extrapolation of cadmium concentration in terrestrial mosses using multiple linear regression model predictions across French biogeographical regions
Source: Environ Sci Pollut Res Int. 2025 Feb 6;32(9):5276–92. doi: 10.1007/s11356-025-35985-5 (PMC11868212; doi:10.1007/s11356-025-35985-5)
Supplement: Supplementary file 1 — Supplementary file1 (PDF 111 KB) [file 11356_2025_35985_MOESM1_ESM.pdf]

Table Supplementary Information

List of variables before and after removing those for which more than 95% of the values were equal to 0, then selection by Spearman correlation; Selected = application of selection of covariates with than 95% of the value equal to 0 in the section ''%0'' and selected covariate with spearman correlation in the section '' Spearman''; Conserved = variables conserved after removing covariates with than 95% of the values equal to 0 and remove if the spearman correlation value exceeds 0.75; Total of variables = total number of variables selected

Variable	Code	variable selection														
		**France (n = 445)**			**Atlantic zone (n = 220)**			**Continental zone (n = 147)**			**Mediterranean zone (n = 49)**			**Alpine zone (n = 29)**		
		**selected**		**conserved**	**selected**		**conserved**	**selected**		**conserved**	**selected**		**conserved**	**selected**		**conserved**
		**% 0**	**Spearman**		**% 0**	**Spearman**		**% 0**	**Spearman**		**% 0**	**Spearman**		**% 0**	**Spearman**	
**Moss species**	**Moss[a]**			✓			✓			✓			✓			✓
**Tree cover**	**Tree[a]**			✓			✓			✓			✓			✓
**Biogeographical zone**	**Biogeo[b]**			✓												
**Altitude**	**Altitude[c]**			✓			✓			✓			✓			✓
**Forested land uses (1, 5, 10, 15 km radius)**	**Forest_[¤d]**		**10, 15**	**1, 5**		**10, 15**	**1, 5**		**10, 15**	**1, 5**		**10, 15**	**1, 5**		**10, 15**	**1, 5**
**Urban land uses (1, 5, 10, 15 km radius)**	**Urban_[¤d]**		**10, 15**	**1, 5**		**10, 15**	**1, 5**		**10, 15**	**1, 5**		**10, 15**	**1, 5**		**10, 15**	**1, 5**
**Industrial land uses (1, 5, 10, 15 km radius)**	**Industrial_[¤d]**	**1**	**15**	**5, 10**	**1**	**15**	**5, 10**	**1**	**10, 15**	**5**	**1**	**15**	**1, 5**	**1**	**15**	**5, 10**
**Pasture land uses (1, 5, 10, 15 km radius)**	**Pasture_[¤d]**		**10, 15**	**1, 5**		**10, 15**	**1, 5**		**10, 15**	**1, 5**		**10, 15**	**1, 5**		**10, 15**	**1, 5**
**Agricultural land uses (1, 5, 10, 15 km radius)**	**Agricultural_[¤d]**		**10, 15**	**1, 5**		**1, 10, 15**	**5**		**10, 15**	**1, 5**		**10, 15**	**1, 5**		**10, 15**	**1, 5**
**Fruits land uses (1, 5, 10, 15 km radius)**	**Fruits_[¤d]**	**1**	**15**	**5, 10**	**1**	**15**	**5, 10**		**10, 15**	**1, 5**		**10, 15**	**1, 5**	**1, 5**	**15**	**10**
**Vegetation land uses (1, 5, 10, 15 km radius)**	**Vegetation_[¤d]**		**10, 15**	**1, 5**	**1**	**15**	**5, 10**		**10, 15**	**1, 5**		**10, 15**	**1, 5**		**1, 5, 10, 15**	
**Water land uses (1, 5, 10, 15 km radius)**	**Water_[¤d]**	**1**	**15**	**5, 10**	**1**	**15**	**5, 10**	**1**	**15**	**5, 10**	**1**		**5, 10, 15**	**1**	**15**	**5, 10**
**Sea land uses (1, 5, 10, 15 km radius)**	**Sea_[¤d]**	**1, 5**	**15**	**10**	**1**	**15**	**5, 10**	**1, 5, 10 , 15**			**1**	**10, 15**	**5**	**1, 5, 10, 15**		
**Railway (1, 5, 10, 15 km radius)**	**Railway_[¤d]**	**1**	**10, 15**	**5**	**1**	**15**	**5, 10**	**1**	**10, 15**	**5**	**1**	**15**	**5, 10**	**1**	**10, 15**	**5**
**Road (1, 5, 10, 15 km radius)**	**Road_[¤d]**	**1**	**10, 15**	**5**	**1**	**10, 15**	**5**	**1**	**10, 15**	**5**	**1**		**5, 10, 15**	**1**	**15**	**5, 10**
**Cd air deposition (1, 3, 6, 9, 12 month)**	**EMEP_dep[+e]**		**6, 9, 12**	**1, 3**		**6, 9, 12**	**1, 3**		**3, 6, 9, 12**	**1**		**6, 9, 12**	**1, 3**		**3, 6, 9, 12**	**1**
**Cd air concentration**	**EMEP_air[e]**			✓			✓			✓			✓			✓
**Cd soil total**	**RMQS_tot[f]**			✓			✓			✓			✓			✓
**Total of variables**				**27**			**27**			**23**			**29**			**22**

[a] Biosurveillance des Retombées Atmosphériques Métalliques par les Mousses, https://bramm.mnhn.fr/
[b] L'Inventaire National du Patrimoine Naturel, https://inpn.mnhn.fr
[c] Copernicus, https://www.copernicus.eu/fr
[d] Corine Land Cover, https://land.copernicus.eu/pan-european/corine-land-cover
[e] European Monitoring and Evaluation Programme, https://www.emep.int/
[f] Réseau de Mesures de la Qualité des Sols, https://www.gissol.fr/le-gis/programmes/rmqs-34
[¤] Represents the length of radius in km
[+] Represents the number of months summed
