# Supplementary material for: Spatial extrapolation of cadmium concentration in terrestrial mosses using multiple linear regression model predictions across French biogeographical regions
Source: Environ Sci Pollut Res Int. 2025 Feb 6;32(9):5276–92. doi: 10.1007/s11356-025-35985-5 (PMC11868212; doi:10.1007/s11356-025-35985-5)
Supplement: Supplementary file 2 — Supplementary file2 (PDF 197 KB) [file 11356_2025_35985_MOESM2_ESM.pdf]

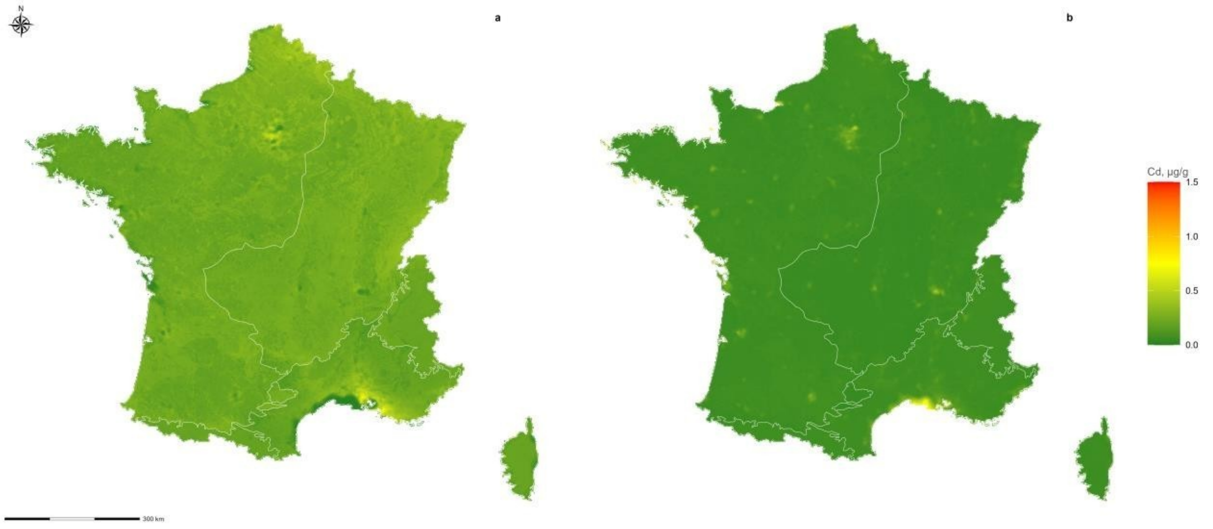

Fig. 1. SI. a Predictions of Cd concentration accumulated by mosses, expressed in *Hypnum cupressiforme* Hedw. under conifers cover tree (µg/g) from the selected 9 covariates model. White lines: border of the biogeographical zones; **b** standard deviation of the prediction from the selected 9 covariates model

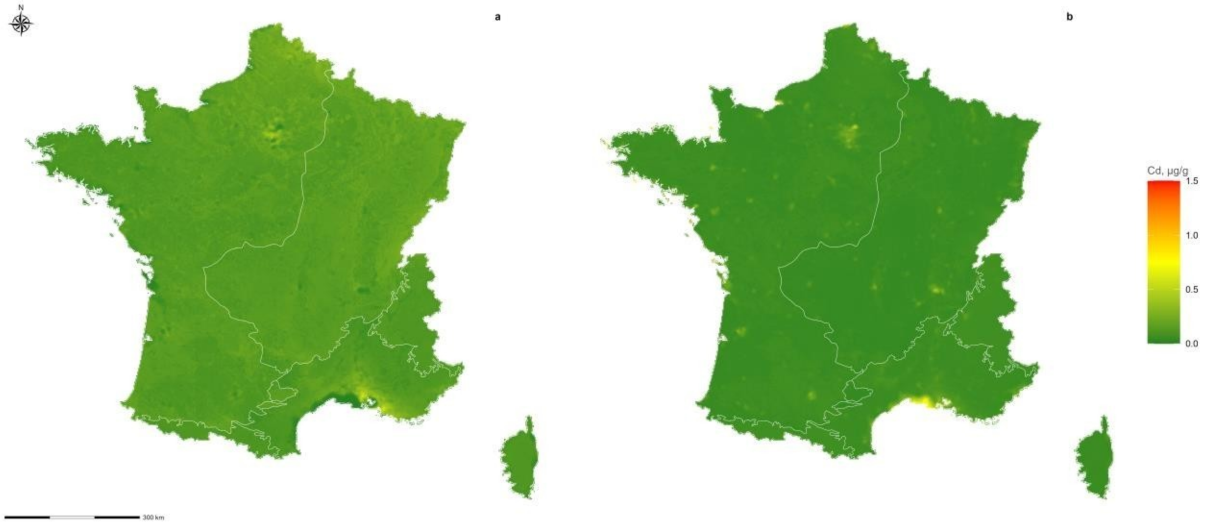

Fig. 2. SI. a Predictions of Cd concentration accumulated by mosses, expressed in *Pseudoscleropodium purum* (Hedw.) M.Fleisch. under conifers cover tree (µg/g) from the selected 9 covariates model White lines: border of the biogeographical zones; **b** standard deviation of the prediction from the selected 9 covariates model

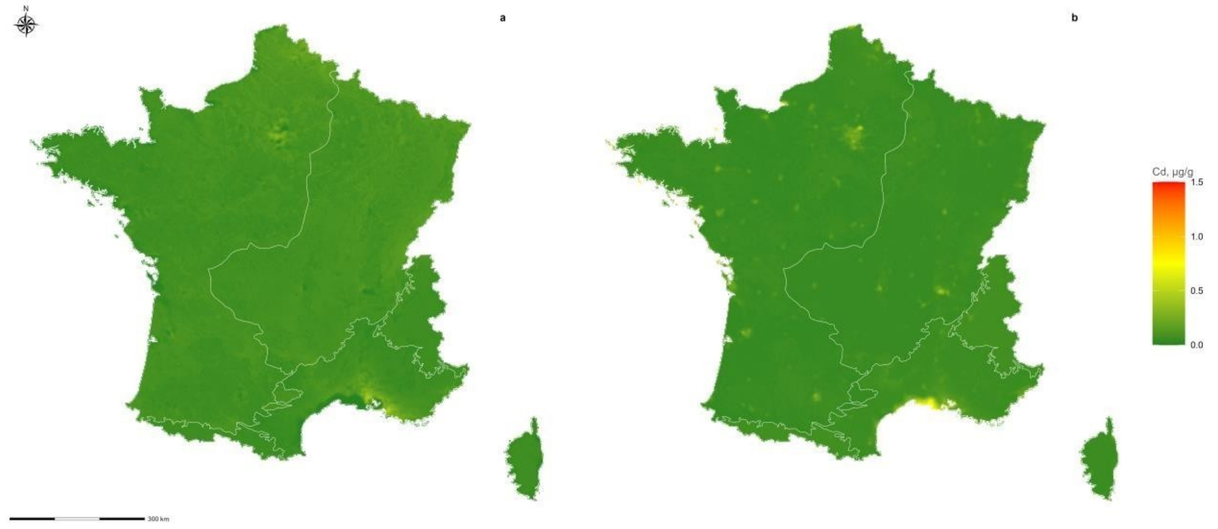

Fig. 3. SI. a Predictions of Cd concentration accumulated by mosses, expressed in *Pseudoscleropodium purum* (Hedw.) M.Fleisch. under deciduous cover tree (µg/g) from the selected 9 covariates model. White lines: border of the biogeographical zones; **b** standard deviation of the prediction from the selected 9 covariates model
